# Parkin–ACSL4 axis in ferroptosis regulation: a narrative review on therapeutic insights from exercise in aging cardiomyocytes

**DOI:** 10.1038/s44324-025-00092-z

**Published:** 2026-01-06

**Authors:** Negin Kordi, Behnam Bagherzadeh-Rahmani, Rezvan KheirAndish, Raheleh Rezaali, Brent R. Stockwell

**Affiliations:** 1 Department of Exercise Physiology, Faculty of Sport Sciences, Razi University, Kermanshah, Iran; 2 Department of Exercise Physiology, Faculty of Sports Sciences, Shomal University, Amol, Iran; 3 Department of Physical Education, Faculty of Humanities and Arts, Technical and Vocational University (TVU), Tehran, Iran; 4 Department of Sports Nutrition and Exercise Physiology, Faculty of Sport Sciences, Central Tehran Branch, Islamic Azad University, Tehran, Iran; 5 Department of Chemistry, Columbia University, New York, NY USA; 6 Department of Biological Sciences, Columbia University, New York, NY USA

**Keywords:** Cardiology, Cell biology, Diseases, Physiology

## Abstract

Ferroptosis, iron-dependent regulated cell death, drives age-related cardiac dysfunction. This review examines aerobic exercise modulation of ferroptosis in aging cardiomyocytes via Parkin–ACSL4 axis. Parkin promotes ACSL4 ubiquitination/degradation, reducing lipid peroxidation and ROS. Exercise activates PINK1/Parkin mitophagy and hepcidin, enhancing mitochondrial resilience and iron homeostasis. Despite promising preclinical evidence, molecular mechanisms remain unclear. Aerobic exercise offers non-pharmacological cardiac protection against ferroptosis in aging.

## Introduction

Aging is a fundamental risk factor for the development of cardiovascular diseases (CVDs), primarily due to cumulative oxidative stress, mitochondrial dysfunction, and disrupted cellular homeostasis^1–3^. These age-associated alterations lead to increased vulnerability of cardiomyocytes, contributing to pathological cardiac remodeling, fibrosis, and heart failure in the elderly population.

Among the various mechanisms contributing to age-related cardiomyocyte injury, ferroptosis, a regulated iron-dependent and non-apoptotic form of cell death, has recently garnered significant attention^4,5^. Distinct from apoptosis and necroptosis, ferroptosis is characterized by iron overload, lipid peroxidation, and mitochondrial damage, including condensed mitochondrial membranes and reduced cristae, while nuclear morphology remains largely preserved^6^. The disruption of this process is linked to the development and advancement of various diseases, including cancer, neurological disorders, acute kidney injury, ischemia-reperfusion, and other related conditions^7,8^. A recent investigation by Liu et al. (2025) proposed that ferroptosis represents a form of regulated cell death driven by intrinsic metabolic dysregulation. Their findings further indicate that the tumor suppressor p53 (a nuclear transcription factor with a pro-apoptotic function) may serve as a key regulator in mediating non-apoptotic cell death pathways, including ferroptosis, necroptosis, pyroptosis, autophagy-dependent cell death, entosis, parthanatos, paraptosis, PANoptosis, cuproptosis, and disulfidptosis^9^. Ferroptosis is biochemically driven by glutathione (GSH) depletion and inactivation of glutathione peroxidase 4 (GPX4), a key antioxidant enzyme responsible for detoxifying lipid peroxides. In the absence of GPX4, ferrous iron (Fe²⁺) participates in Fenton-like reactions, amplifying reactive oxygen species (ROS) and ultimately triggering cell death^10^. Additionally, the enzyme acyl-CoA synthetase long-chain family member 4 (ACSL4) plays a critical role in determining ferroptosis sensitivity by facilitating the incorporation of polyunsaturated fatty acids (PUFAs) into membrane phospholipids, thereby promoting lipid peroxidation^11^.

Cardiomyocytes are particularly susceptible to ferroptosis due to their high oxygen consumption, elevated mitochondrial iron content, and dependence on oxidative phosphorylation. Iron dysregulation frequently observed in aging, myocardial infarction, and ischemia reperfusion (I/R) injury further sensitizes these cells to ferroptotic stress. Recent studies have implicated ferroptosis in myocardial fibrosis, ventricular remodeling, and cardiomyopathy, identifying it as a promising target in age-related heart disease^12^.

As reported by Xiao et al. (2025), Parkin, a crucial factor, is inhibited in cardiomyocytes subjected to iron overload. Recent evidence suggests that Parkin may counteract iron-overload-induced ferroptosis in cardiomyocytes, potentially by promoting ACSL4 ubiquitination or degradation through indirect mechanisms^13^. A key regulator of mitochondrial quality control is Parkin, an E3 ubiquitin ligase that activates mitophagy via the PTEN-induced kinase 1 (PINK1)/Parkin signaling pathway. In aged or stressed cardiomyocytes, reduced Parkin expression contributes to impaired mitophagy and mitochondrial dysfunction. Intriguingly, Parkin has been shown to suppress ferroptosis by ubiquitinating and degrading ACSL4, thereby attenuating lipid peroxidation and protecting cell viability^13^. Experimental data indicate that p53 might transcriptionally repress Parkin expression under iron-overload conditions, thereby enhancing ferroptotic vulnerability^14^.

Aerobic exercise, a well-established cardioprotective intervention, exerts beneficial effects on mitochondrial function, oxidative stress, iron metabolism, inflammation, and overall cellular resilience. It enhances mitophagy through PINK1/Parkin activation and contributes to iron homeostasis by modulating hepcidin expression and systemic metabolism^15,16^. These multifaceted effects suggest that aerobic exercise may mitigate ferroptotic damage in aging cardiomyocytes. Huang et al. (2024) observed reduced ACSL4 expression in skeletal muscle following exercise^17^, whereas Liu et al.^18^ did not observe such changes in cardiac tissue. This discrepancy may reflect tissue-specific metabolic responses or differences in experimental design.

Many studies to date have focused on young animal models, which may not adequately reflect age-related susceptibility to ferroptosis. Given the complex interplay between iron overload, mitophagy, lipid peroxidation, and ferroptosis, aerobic exercise may serve as a powerful non-pharmacological strategy to combat cardiac aging. However, the precise molecular mechanisms linking exercise-induced Parkin activation, ACSL4 modulation, and ferroptosis suppression in aging cardiomyocytes remain unclear. Addressing this gap may provide new insights into non-pharmacological interventions for cardiac aging.

## Pathophysiology of ferroptosis

### Mechanism and morphology

Morphologically, this process is primarily observed in cells exhibiting a reduction in mitochondrial volume, an increase in bilayer membrane density, and a decrease or complete loss of mitochondrial cristae^19,20^; notably, the cell membrane remains intact. The nucleus retains its typical size, and there is an absence of chromatin condensation. On a biochemical level, ferroptosis is facilitated by a depletion of intracellular GSH and a decrease in the activity of GPX4. The reduction reaction catalyzed by GPX4 is unable to metabolize lipid peroxides, which permits Fe^2+^ to oxidize lipids in a manner akin to the Fenton reaction, leading to the generation of significant quantities of ROS^20,21^.

From a genetic perspective, ferroptosis is a process governed by multiple genes^22^. The inhibition of ferroptosis can be achieved through the use of lipophilic antioxidants, chelators, inhibitors of lipid peroxidation, and reduced levels of polyunsaturated fatty acyl phospholipids (PUFA-PLs), which are the primary substrates involved in lethal lipid peroxidation^4,23^. Ferroptosis is characterized by iron-dependent lipid peroxidation rather than a specific Fe/S ratio. While excess intracellular iron amplifies oxidative stress via Fenton chemistry, sulfur-containing antioxidants such as glutathione and thioredoxin act as counter-regulatory elements. The main cause of oxidative stress is iron^24^, and proteins (such as thioredoxin) and peptides (such as glutathione) provide the primary resistance to oxidative stress through S or sulfhydryl’s (SH-) groups^25^. Excessive iron or insufficient sulfur levels play a significant role in the process of ferroptosis. A crucial element of this mechanism involves the iron-mediated peroxidation of unsaturated fatty acids found in phospholipids. The impairment of cellular glutathione-dependent antioxidant defenses initiates ferroptosis, leading to the buildup of harmful lipid reactive oxygen species^19,26^. GSH primarily functions as a redox buffer and cofactor for GPX4 rather than a major cytosolic iron ligand. However, GSH indirectly modulates intracellular iron redox cycling by maintaining Fe²⁺ in a reduced but less reactive state^27^. GSH is a critical substrate for GPX4. GPX4 detoxifies membrane lipid peroxides, thereby preventing the initiation of ferroptotic death^28,29^.

### Ferroptosis inducers and inhibitors

Several small molecules and environmental cues have been identified that either promote or inhibit ferroptosis by targeting key components of its biochemical pathway.

Erastin is recognized as one of the earliest compounds that induce ferroptosis among the inducers. It acts by blocking the cystine/glutamate antiporter system Xc⁻, consequently leading to a decrease in intracellular GSH, a vital antioxidant that shields cells from lipid peroxidation^19^. Another potent inducer, RSL3, directly inhibits GPX4, an essential enzyme that detoxifies lipid peroxides, thereby allowing toxic lipid ROS to accumulate^20^. FIN56 (a type 3 ferroptosis inducer) induces ferroptosis through two mechanisms: it promotes GPX4 degradation and simultaneously depletes coenzyme Q10, a lipid-soluble antioxidant in the mitochondrial membrane^30^. FINO2 (a ferroptosis-inducing peroxide compound) promotes lipid peroxidation by oxidizing labile Fe²⁺ and indirectly compromising GPX4-dependent antioxidant defense, rather than directly inhibiting the enzyme^31^.

On the other hand, several compounds have shown promise in inhibiting ferroptosis. Ferrostatin-1 and liproxstatin-1 are synthetic radical-trapping agents that specifically scavenge lipid ROS and preserve membrane integrity^21,32^. Natural antioxidants such as vitamin E (α-tocopherol) also inhibit lipid peroxidation due to their lipid solubility and chain-breaking antioxidant properties^27^. Additionally, Pharmacological or genetic inhibition of ACSL4 reduces PUFA incorporation into phospholipids, thereby decreasing lipid peroxidation susceptibility in experimental models^33^.

The composition of membrane fatty acids also critically determines ferroptotic susceptibility. Membranes enriched in PUFAs, such as arachidonic acid (AA) and adrenic acid (AdA), are highly prone to peroxidation and thus promote ferroptosis. In contrast, monounsaturated fatty acids (MUFAs) such as oleic acid can replace oxidizable PUFA chains in phospholipids, thereby conferring resistance to ferroptosis^34^. The balance between PUFA and MUFA content plays an essential role in modulating cardiomyocyte vulnerability to ferroptotic death, especially under stress or aging conditions.

### Mechanism of ferroptosis in cardiomyocytes

Research has indicated that the Modulation rather than inhibition of mitochondrial autophagy appears critical for optimal cardiac recovery after myocardial infarction, since both insufficient and excessive mitophagy can exacerbate myocardial injury^29^. PINK1, a serine/threonine kinase targeted to mitochondria, along with Parkin, a cytoplasmic ubiquitin E3 ligase, plays a crucial role in mitochondrial autophagy when mitochondria sustain damage. The reduction of mitochondrial membrane potential results in the accumulation of PINK1 within the outer mitochondrial membrane^35^, where PINK1 facilitates the phosphorylation of serine 65 (Ser65) in the ubiquitin-like domain of Parkin, thereby activating and recruiting Parkin^36–38^, which subsequently triggers mitochondrial autophagy.

Studies have shown that the long non-coding RNA H19 suppresses the PINK1/Parkin pathway, thereby mitigating excessive mitochondrial autophagy induced by palmitic acid. This action subsequently enhances mitochondrial respiration and Adenosine triphosphate (ATP) production in myocardial cells^39^. Consequently, the mechanism underlying myocardial injury and fibrosis following myocardial infarction may be linked to the excessive mitochondrial autophagy that is mediated by the PINK1/Parkin pathway^27^.

Therefore, ferroptosis is intricately linked to the mechanism of myocardial fibrosis; however, it remains uncertain whether ferroptosis in myocardial cells is connected to excessive mitochondrial autophagy and plays a role in the development of myocardial fibrosis post-ischemia. Hydrogen sulfide (H_2_S) is a gasotransmitter that was identified after carbon monoxide (CO) and nitric oxide (NO)^40^. Evidence is accumulating that H₂S is involved in significant pathological mechanisms related to cardiovascular diseases, including hypertension, the formation of atherosclerotic plaques, dysregulated angiogenesis, and ischemic heart injury^41^. H_2_S serves a protective function for the cardiovascular system and is linked to antioxidant, anti-apoptotic, anti-inflammatory, and antifibrotic properties^42^. Research has demonstrated that the H₂S donors such as NaHS generally exert anti-fibrotic effects in cardiac and metabolic models by reducing oxidative stress and inhibiting fibroblast activation^43^ and diabetes^13^. Nonetheless, emerging evidence suggests that mitochondria-targeted H₂S donors such as AP39 (a Mitochondria-Targeted Hydrogen Sulfide Donor) may attenuate post-infarction fibrosis by suppressing mitochondrial ROS and ferroptotic signaling^44^.

### Clinical relevance

A growing body of clinical and experimental evidence supports the critical role of iron overload in the pathogenesis of cardiac injury. In myocardial infarction (MI) and ischemia/reperfusion (I/R) injury, tissue iron levels surge, amplifying oxidative damage and triggering ferroptotic pathways^45^. Iron-mediated lipid peroxidation during reperfusion contributes significantly to cardiomyocyte death, alongside other regulated death pathways such as apoptosis and necroptosis, underscoring ferroptosis as a central but not exclusive mechanism.

For example, administration of deferoxamine (DFO) a clinically used iron chelator has been demonstrated to reduce infarct size, preserve mitochondrial function, and suppress ferroptosis in rodent models of I/R injury^46,47^. Notably, iron overload in aging is often systemic, with iron deposition observed in the heart, liver, brain, and vascular system, thereby linking it to multi-organ dysfunction^48^.

These findings highlight the translational potential of modulating iron metabolism as a therapeutic approach. Pharmacological agents that target iron transport, storage, or chelation alongside lifestyle interventions such as aerobic exercise, which has been shown to modulate systemic iron levels, could offer novel strategies for attenuating ferroptosis and preserving cardiac function in older adults^49^.

### Role of iron in cardiomyocyte ferroptosis

Iron homeostasis in cardiomyocytes is tightly regulated by an intricate network of iron transporters, storage proteins, and redox modulators. Transferrin receptor 1 (TfR1) facilitates cellular iron uptake by binding to circulating transferrin-bound iron and internalizing it via endocytosis. In aging myocardium, TfR1 expressions may be altered in aging myocardium, though evidence remains inconsistent across studies, potentially reflecting tissue heterogeneity or compensatory changes in iron metabolism^50^.

Once inside the cell, iron is either utilized in metabolic processes or sequestered by ferritin, the primary iron storage protein. However, under conditions of iron overload, ferritin capacity becomes saturated, and the surplus iron accumulates as redox-active Fe²⁺, which catalyzes Fenton reactions and generates hydroxyl radicals, exacerbating lipid peroxidation and oxidative stress^51,52^.

Experimental models often employ In vitro exposure to ferric ammonium citrate (FAC) is a commonly used model to induce ferroptosis-like changes in cardiomyocytes, though it represents supra-physiological iron levels not fully reflective of in vivo conditions^53^. Excess iron directly disrupts mitochondrial dynamics and bioenergetics, impairing membrane potential and respiratory chain activity. These alterations contribute to heightened susceptibility to ferroptosis, particularly in aged or metabolically stressed cardiomyocytes^54^.

### The role of parkin in cardiomyocytes’ ferroptosis

Gustafsson’s research team carried out the first thorough in vivo study on heart function in mice completely lacking the parkin gene. Interestingly, these mice didn’t show the expected neurological issues seen in other parkin-deficiency models^55^, nor did they initially display any heart dysfunction or structural abnormalities^56^. This could mean that other backup mechanisms for clearing damaged mitochondria (mitophagy) compensate for the loss of parkin in the heart and other tissues. Alternatively, parkin might simply not play a major role in maintaining mitochondrial health in the heart or it could be a mix of both factors^57^.

What makes this especially intriguing is that heart muscle is packed with mitochondria (making up 30–40% of the heart’s weight), and keeping these mitochondria healthy is crucial to preventing toxicity from damaged or aging ones^58^. Yet, despite this heavy reliance on mitochondria, normal adult mouse hearts have very little parkin protein^56^, suggesting that other quality-control systems might be doing most of the work.

Excessive iron accumulation heightens the likelihood of cardiovascular conditions, including hereditary hypopigmentation, myocardial infarction and cardiac ischemia/reperfusion injury^13^. The main contributor to myocardial damage resulting from iron overload is oxidative stress^44^. Within cardiac tissue, oxidative stress disrupts excitation–contraction coupling, harms cell and mitochondrial membranes via lipid peroxidation, obstructs mitochondrial oxidative phosphorylation and ATP production, and leads to damage of both Deoxyribonucleic acid (DNA) and mitochondrial DNA (mtDNA)^59^. Iron is said to enhance lipid peroxidation via the Haber‒Weiss and Fenton reactions^59^. Nevertheless, research suggests that iron influences lipid metabolism through the p53–Parkin–ACSL4 pathway, indicating that the role of iron in ferroptosis is governed by various proteins rather than being limited to chemical reactions alone. Furthermore, excess iron exacerbates myocardial infarction, and the inhibition of iron or ferroptosis has been shown to reduce myocardial injury^13^.

Under physiological conditions, Parkin expression in the adult heart is relatively low but markedly upregulated in response to mitochondrial or oxidative stress^13,60^. Parkin directs cardiac metabolic maturation and the elimination of mitochondria during the perinatal phase^61,62^. The targeted deletion of Park2, encoding Parkin in cardiomyocytes, impairs postnatal mitochondrial maturation and does not affect Mitofusin-2 (MFN2) regulation of mitophagy, resulting in rapid mortality in most mice. In response to I/R injury, Parkin transgenic mice show enhanced cardiac remodeling and function through the suppression of mitochondrial permeability transition pore (mPTP) opening by facilitating the ubiquitination of cyclophilin D (CypD) during necrotic processes^63^. Furthermore, Parkin transgenic mice reduce doxorubicin (DOX)-induced cardiotoxicity by enhancing mitophagy and inhibiting apoptosis^63^. Interestingly, it has been demonstrated that Experimental studies suggest that Parkin overexpression mitigates iron overload induced injury and ischemia-related ferroptotic signaling in rodent models, though confirmation in human cardiac tissue remains pending. Moreover, studies conducted on animals have shown that the overexpression of Parkin alleviates myocardial injury caused by I/R. Significantly, mice with cardiac-specific Parkin knockout induced by iron overload show elevated levels of tissue ferroptosis. Therefore, the Parkin protein could represent a new therapeutic target for cardiomyopathy^13^.

According to Xiao et al. (2025), iron excess caused mitophagy to decline, although Parkin overexpression encouraged mitophagy to prevent ferroptosis. These findings point to a possible connection between ferroptosis and mitophagy. According to earlier research, ferroptosis and mitophagy may be related. For instance, ferritinophagy enhances ferroptotic sensitivity by liberating iron from ferritin stores, expanding the labile iron pool that fuels lipid peroxidation^13^. Ferroptosis contributes to vascular aging by causing a reduction in NAD^+^ levels and facilitating NCOA4-mediated ferritinophagy^64^. Mitophagy results in the lysosomal breakdown of lipid droplet mitochondria, the liberation of free fatty acids, and a notable rise in lipid peroxidation, thereby enhancing Ferroptosis^65^. Conversely, mitophagy might result in the entrapment of iron within mitophagosomes, consequently diminishing the availability of ROS necessary for ferroptosis^66^. The interplay between mitophagy and ferroptosis is intricate, and the prospective function of Parkin in the mediation of ferritinophagy requires additional exploration in the future^13^.

#### The role of Parkin and the PINK1 pathway in ferroptosis regulation

Although Parkin is scarcely expressed in healthy adult hearts, its levels rise significantly under various forms of cardiac stress. Studies have shown that Parkin protein expression increases within 8 h post-myocardial infarction and remains elevated for at least 48 h in the infarct border zone of mouse hearts^56,67^. A similar upregulation occurs at both the mRNA and protein levels following the disruption of mitochondrial fission in cardiomyocytes, achieved through cardiac-specific genetic inactivation of dynamin-related protein 1 (Drp1)^68,69^. Additionally, PINK1 deficiency disrupts Parkin recruitment to damaged mitochondria, impairing mitophagy rather than enhancing Parkin protein levels^56,70^. These observations collectively suggest that Parkin induction is a generalized response to mitochondrial stress, even in the absence of overt cardiac dysfunction. However, the pronounced increase in Parkin levels in dynamin-related protein 1 (Drp1)-deficient and PINK1-deficient hearts contrasted with normal controls was not always easily discernible in some original study figures. Nevertheless, immunoblot analyses with standardized protein loading across all groups confirm this upregulation^60,67^. Functionally, stress-induced Parkin has been linked to pathological mitophagy. This is supported by the reversal of hyperactivated mitophagy and Drp1 ablation-induced cardiomyopathy upon cardiomyocyte-specific Parkin deletion^56^. Moreover, germline Parkin ablation impairs compensatory mitophagy, which normally clears damaged mitochondria from peri-infarct regions after cardiac ischemia^56^.

The PINK1–Parkin signaling axis plays a pivotal role in mitochondrial quality control through the process of mitophagythe selective degradation of damaged mitochondria. Under homeostatic conditions, the serine/threonine kinase PINK1 is imported into healthy mitochondria and rapidly degraded. However, upon mitochondrial depolarization or oxidative injury, PINK1 accumulates on the outer mitochondrial membrane (OMM), where it recruits and activates the E3 ubiquitin ligase Parkin^71^.

Activated Parkin ubiquitinates a range of outer mitochondrial membrane proteins, tagging damaged mitochondria for degradation via autophagosomes. This mechanism mitigates ROS accumulation by eliminating dysfunctional mitochondria, thereby reducing a major source of oxidative stress and protecting against ferroptosis^72^.

Intriguingly, Parkin also regulates ferroptosis through a non-canonical pathway involving ACSL4, a key pro-ferroptotic enzyme. Emerging evidence indicates that Parkin may modulate ACSL4 stability via ubiquitination in certain cell types, though direct confirmation in cardiomyocytes requires further investigation^73^.

Moreover, Parkin contributes to the maintenance of mitochondrial dynamics and bioenergetic efficiency. In stressed cardiomyocytes, preserved mitochondrial integrity is crucial to prevent the metabolic collapse that predisposes cells to ferroptosis^74^.

A notable negative regulator of this pathway is p53, which transcriptionally represses Parkin. Under conditions of iron overload, p53 is activated and suppresses Parkin expression, thereby attenuating mitophagy, enhancing ACSL4 accumulation, and sensitizing cardiomyocytes to ferroptotic death^75^.

#### Interplay between Parkin, Iron, and ACSL4

A complex but highly coordinated regulatory circuit exists between iron metabolism, ACSL4 activity, and Parkin-mediated mitophagy, forming a critical checkpoint for ferroptosis susceptibility.

Excess intracellular iron increases the labile iron pool (LIP), which fuels Fenton chemistry and lipid ROS generation. Concurrently, elevated iron levels have been shown to upregulate ACSL4, enhancing PUFA incorporation into membrane phospholipids, thus increasing the substrate pool for lipid peroxidation^76^.

Parkin serves as a protective counterbalance in this environment. Through mitophagy, it removes damaged mitochondria, which often accumulate iron, and via ubiquitination of ACSL4, Parkin suppresses the initiation of ferroptosis^77^.

This dynamic interaction forms a feedback loop: iron overload accelerates ferroptosis via ACSL4, while Parkin activation either through exercise, genetic overexpression, or pharmacologic agents interrupts this cascade. The therapeutic implication is significant: Modulating the Parkin–ACSL4–iron regulatory axis represents a promising, yet still experimental, therapeutic target for ferroptosis-related cardiac protection^78^.

**Fig. 1:**
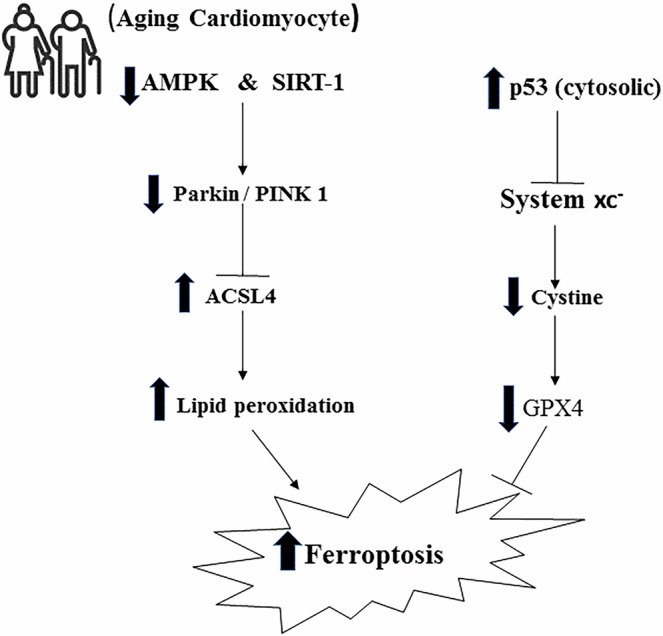
Schematic illustration of the molecular pathways regulating ferroptosis in aging cardiomyocytes. During cardiomyocyte aging, impaired AMPK/SIRT1 signaling suppresses the PINK1–Parkin pathway, leading to reduced mitophagy and increased ACSL4 expression. Elevated ACSL4 promotes the incorporation of polyunsaturated fatty acids into membrane phospholipids, enhancing lipid peroxidation and ferroptotic susceptibility. Meanwhile, cytosolic p53 inhibits the cystine/glutamate antiporter (System Xc⁻), thereby limiting cystine uptake and glutathione synthesis. The resulting decrease in GPX4 activity impairs antioxidant defense, promotes lipid peroxide accumulation, and contributes to ferroptosis.

The cellular mechanisms of ferroptosis in cardiomyocytes are illustrated in Fig. 1.

## Exercise as a modulator of ferroptosis in aging

### The impact of exercise on Iron Homeostasis

Exercise plays a significant regulatory role in systemic and cellular iron metabolism. One well-characterized mechanism is the upregulation of hepcidin, a liver-derived peptide hormone that inhibits intestinal iron absorption and macrophage iron release. During acute bouts of exercise, transient IL-6 elevation induces short-term hepcidin synthesis, temporarily lowering plasma iron. However, with regular training, this response becomes attenuated, helping maintain systemic iron balance over time^79^. Moreover, Chronic aerobic training modulates ferritin and transferrin receptor 1 (TfR1) expression in cardiomyocytes, maintaining optimal intracellular iron balance rather than simply minimizing iron content. This adaptive regulation prevents excessive labile iron accumulation while preserving sufficient stores for metabolic needs^80,81^. Exercise also enhances the expression of ferroportin, the sole known iron exporter, promoting iron efflux and maintaining cellular iron balance^82^. Additionally, exercise-induced anti-inflammatory signaling, together with vitamin D modulation, contributes indirectly to iron homeostasis by regulating hepcidin and redox pathways^83^. Collectively, these adaptations reduce iron-mediated susceptibility to ferroptosis in aging cardiac tissue.

### The impact of exercise on amino acid and lipid metabolism

Physical activity promotes mitochondrial bioenergetics and shifts metabolic substrate preference, thereby lowering the risk of lipid peroxidation. Exercise coordinately modulates signaling pathways, including transient activation of Mammalian/mechanistic target of rapamycin (mTOR) for protein synthesis and sustained induction of PGC-1α for mitochondrial biogenesis and fatty acid oxidation^84,85^. Upregulation of mitochondrial transcription factor A (TFAM) ensures mitochondrial DNA replication and maintenance, preserving mitochondrial integrity^86^. Furthermore, exercise reduces visceral fat mass and improves circulating lipid profiles, optimizing the PUFA/ saturated fatty acid (SFA) ratio and thereby reducing the pool of peroxidation-prone lipid species^87^. Exercise also supports GSH synthesis by increasing cysteine bioavailability and stimulating cystine/glutamate exchange, thereby maintaining GPX4 activity and enhancing antioxidant capacity^88^. These metabolic adjustments collectively reinforce cellular resistance to oxidative lipid damage and ferroptotic death.

### Exercise-induced parkin activation

Exercise can enhance Parkin expression and activate the PINK1–Parkin mitophagy pathway, particularly under endurance or moderate-intensity regimens that promote mitochondrial turnover^89^. Acute aerobic exercise promotes Parkin translocation to damaged mitochondria, facilitating their mitophagic clearance and reducing ROS production^15^. Long-term training sustains Parkin upregulation, improving mitochondrial turnover and bolstering cellular defenses against ferroptotic stressors^90^. In animal models, treadmill or voluntary wheel running reverses aging-associated decline in Parkin expression, rejuvenating mitochondrial quality control mechanisms^91^. This exercise-induced mitophagy is critical for maintaining cardiac resilience and attenuating ferroptosis in aged myocardium.

### Interaction of exercise, parkin, and ferroptosis

The interaction between exercise, Parkin signaling, and ferroptosis inhibition constitutes a potential protective feedback loop. Exercise-induced Parkin activation enhances mitophagy, which clears damaged mitochondria and lowers ROS generation. Concurrently, exercise may downregulate ACSL4 expression or activity indirectly through reduced oxidative stress and improved lipid metabolism, thereby lowering susceptibility to lipid peroxidation^62^. Additionally, exercise elevates GPX4 and glutathione levels, strengthening the antioxidant defense system^92^. Together, these molecular events improve cardiac function, decrease oxidative injury, and increase stress tolerance in aging cardiomyocytes. Therapeutic strategies that mimic exercise effects or target this axis may offer promising avenues to prevent age-related cardiovascular decline.

**Fig. 2:**
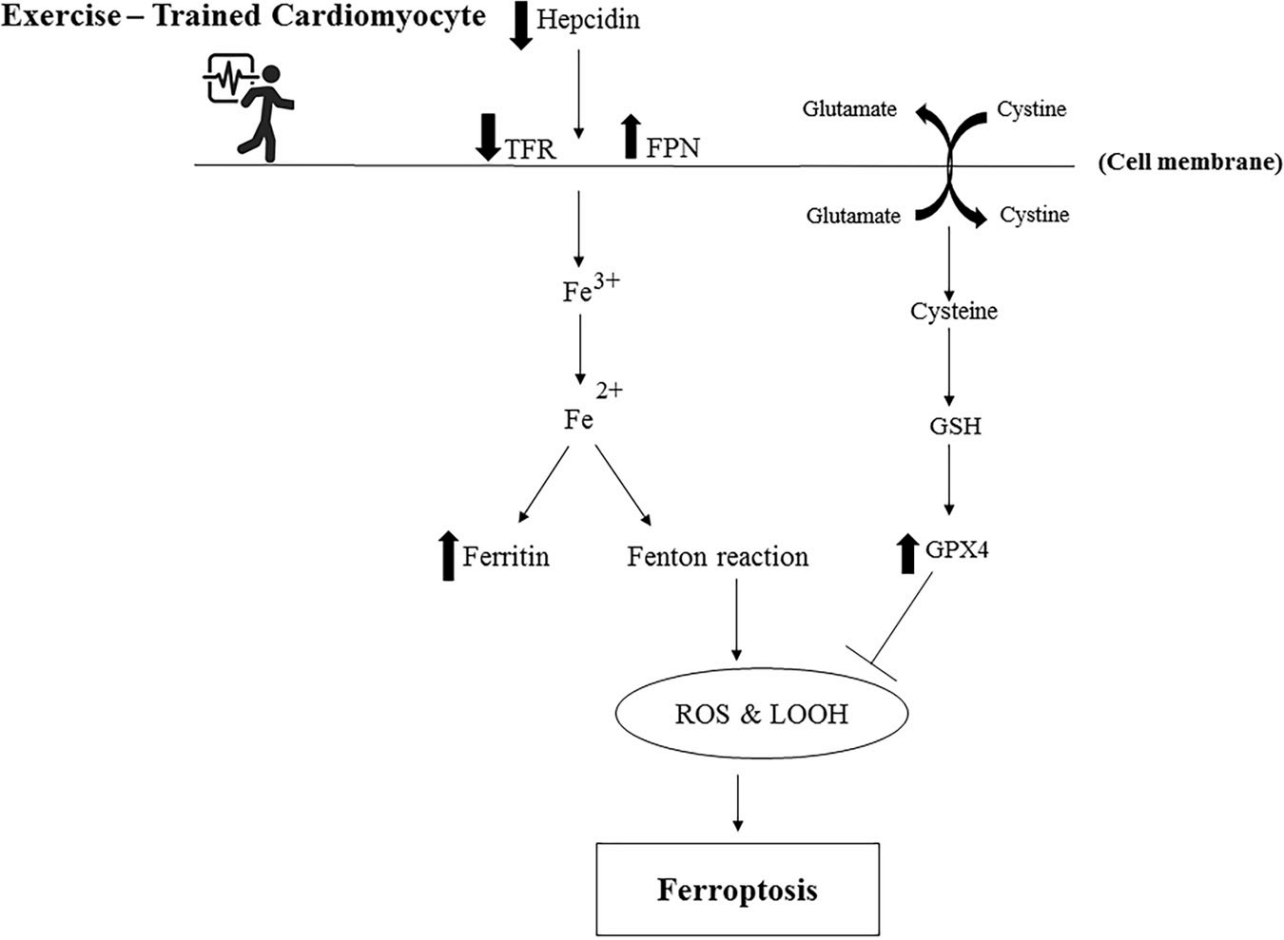
Exercise-induced inhibition of ferroptosis signaling pathways in cardiomyocytes. Aerobic training suppresses ferroptosis in cardiomyocytes by regulating iron metabolism and antioxidant defense systems. Reduced hepcidin expression decreases transferrin receptor (TFR) levels and enhances ferroportin (FPN) activity, limiting intracellular iron accumulation and Fenton reaction–mediated ROS generation. Concurrently, exercise elevates cysteine uptake and glutathione (GSH) synthesis, promoting GPX4 activity that detoxifies lipid peroxides. Together, these adaptations attenuate oxidative stress and lipid peroxidation, thereby protecting cardiomyocytes from ferroptotic cell death.

Exercise-induced inhibition of ferroptosis signaling pathways is illustrated in Fig. 2.

### Dual modulatory pathways of ferroptosis: aging versus exercise

Ferroptosis in cardiomyocytes is governed by a dynamic balance between deleterious stimuli linked to aging and protective adaptations induced by physical activity. Aging is associated with progressive iron accumulation and dysregulation of lipid metabolism, resulting in a cellular milieu prone to ferroptotic death. Elevated intracellular iron levels promote oxidative stress, which can secondarily induce ACSL4 expression, enhancing PUFA incorporation into membrane phospholipids. These PUFA-enriched membranes are highly susceptible to peroxidation, especially under heightened ROS conditions. The ensuing lipid peroxides compromise mitochondrial function, triggering ferroptosis and cardiomyocyte loss, which contribute to myocardial fibrosis and diminished cardiac performance in older adults^93,94^.

Conversely, regular aerobic exercise enhances the expression of Parkin, a key E3 ubiquitin ligase involved in mitophagy. Activation of the PINK1–Parkin axis promotes the selective removal of dysfunctional, ROS-generating mitochondria rich in iron. Simultaneously, exercise suppresses ACSL4 expression, limiting the pool of peroxidation-prone lipids in cardiomyocyte membranes. These coordinated adaptations reduce lipid peroxidation, preserve mitochondrial function, and protect cardiac cells from ferroptotic death^95,96^.

This dual-pathway model underscores the contrasting influences of aging and exercise on ferroptosis regulation and highlights the therapeutic potential of lifestyle interventions to improve cardiovascular health in aging populations (see Table 1).

**Table 1:** Dual modulatory effects of aging and exercise on ferroptosis-related pathways in cardiomyocytes

Pathway / Factor	Effect of Aging	Effect of Exercise	Main Mechanism (Proposed / Supported Evidence)
Labile Iron Pool (Fe²⁺)	Increases	Normalizes or decreases	Exercise promotes systemic iron regulation via transient hepcidin response, enhanced ferroportin expression, and reduced oxidative iron accumulation.
ACSL4 Expression / Activity	Upregulated	Potentially downregulated (indirect evidence)	Exercise may indirectly modulate ACSL4 through improved lipid metabolism and reduced oxidative stress; direct regulation remains to be confirmed.
GPX4 / GSH System	Reduced activity	Enhanced activity	Exercise enhances antioxidant defenses by increasing cysteine availability and maintaining GPX4-dependent detoxification of lipid peroxides.
Parkin / PINK1 Pathway	Downregulated	Upregulated	Exercise activates the PINK1–Parkin mitophagy pathway, supporting mitochondrial quality control and reducing ROS accumulation.
Lipid Peroxidation / ROS	High accumulation	Reduced	Exercise improves mitochondrial turnover and antioxidant capacity, limiting ROS-driven lipid peroxidation.
Ferroptosis Sensitivity	Increases	Decreases	Exercise-induced mitochondrial maintenance and redox balance confer greater resistance to ferroptotic stress.

## Discussion

Growing evidence suggests that ferroptosis plays a crucial role in age-related heart problems. However, the regulatory effects of exercise on ferroptosis remain largely hypothetical, with limited direct evidence available. This review aims to integrate emerging insights that may link physical activity to ferroptosis modulation in aging cardiomyocytes.

It is interesting to consider that exercise might affect how vulnerable the aging heart is to ferroptosis. Regular exercise can influence several factors that lead to ferroptosis, such as oxidative stress, iron balance, and lipid peroxidation. For example, exercise may activate pathways that defend against damage and help rejuvenate mitochondria. This could reduce the oxidative environment that makes cells more likely to undergo ferroptosis^97^. Exercise enhances Parkin expression, improves mitochondrial dynamics, and modulates systemic iron homeostasis through a transient increase in hepcidin during acute exercise which may provide short-term protective effects by limiting labile iron and through long-term adaptations, including regulation of ferroportin-mediated iron export. Chronic aerobic exercise helps fine-tune hepcidin levels, preventing excessive iron accumulation while maintaining adequate iron availability. These multifaceted effects position exercise as a promising strategy to reduce ferroptosis-related cardiomyocyte damage and delay age-associated cardiovascular decline (Fig. 2).

Parkin, a well-established regulator of mitophagy, could indirectly influence ferroptosis by maintaining mitochondrial integrity and redox balance. While most evidence comes from neuronal or hepatic studies, its potential cardiac role warrants investigation^98^. Recent evidence highlights mitochondria as both a source and a regulatory hub of ferroptotic signaling in cardiomyocytes. Beyond iron handling and ROS generation, mitochondrial dysfunction directly contributes to lipid peroxidation and metabolic collapse during cardiac aging. Parkin, an E3 ubiquitin ligase, directly interacts with and ubiquitinates ACSL4 via the lysine 48 (K48)-linked polyubiquitination, targeting it for proteasomal degradation and thereby reducing ACSL4 protein levels. ACSL4 facilitates the incorporation of PUFAs like arachidonic acid into phosphatidylethanolamine (PE) phospholipids, rendering them prone to peroxidation and promoting ferroptosis. By limiting ACSL4 activity, Parkin decreases PUFA-PE content, suppresses lipid ROS accumulation, and inhibits ferroptotic lipid peroxidation, as demonstrated in iron overload models in cardiomyocytes (Fig. 1)^13^. Recent studies demonstrate that impaired mitophagy, defective mitochondrial cristae remodeling, and altered iron–sulfur cluster biogenesis amplify ferroptosis susceptibility. Parkin-mediated mitophagy acts as a key defense mechanism by removing dysfunctional mitochondria and preventing the release of excessive ROS and iron from damaged organelles^99–102^. Thus, the Parkin–ACSL4 axis likely operates in close coordination with mitochondrial quality control systems to preserve cardiomyocyte survival during aging. Exercise-induced activation of Parkin not only restores mitophagic flux but also indirectly limits ACSL4-dependent lipid remodeling, establishing mitochondria as a pivotal therapeutic target in ferroptosis regulation^17,101,103^.

On the other hand, although direct evidence on exercise-mediated regulation of ACSL4 in cardiac tissue is lacking, existing data on exercise-induced lipid remodeling suggests potential indirect modulation of this enzyme’s activity. We still don’t know if exercise can reduce the expression or activity of ACSL4 in the aging heart, which is a question that future therapies may explore^17^.

Importantly, the existing research on this topic is still developing, with most evidence coming from animal studies. These methodological differences hinder the ability to draw consistent and translationally relevant conclusions regarding the effects of exercise on ferroptosis. Additionally, ferroptosis is a complex process, influenced by various metabolic, redox, and mitochondrial signals, making it hard to identify clear mechanistic links.

Nonetheless, the intersection of exercise biology and ferroptosis signaling holds great promise. Understanding how exercise influences these signaling pathways could help develop non-drug strategies to slow down heart aging or improve the resilience of heart cells. Future studies will need to identify specific molecular targets of exercise, understand the dose-response relationships, and investigate potential interactions with other aging processes like inflammation and mitochondrial dysfunction. Also, Future studies should examine whether endurance and resistance training have differential effects on ACSL4 expression in aging myocardium.

## Conclusion

Ferroptosis represents a critical and distinct mode of regulated cell death that is increasingly recognized as a key contributor to age-related cardiac dysfunction. In aging cardiomyocytes, dysregulated iron homeostasis, impaired antioxidant defenses, and aberrant lipid metabolism converge to create a cellular environment that favors ferroptotic cell death. Our synthesis of current literature highlights the central role of iron overload and ACSL4-mediated lipid peroxidation in driving ferroptosis in cardiomyocytes, particularly under ischemic or oxidative stress conditions^97^.

Emerging evidence positions Parkin, a crucial regulator of mitochondrial quality control, as a protective modulator against ferroptosis. By promoting mitophagy and potentially influencing lipid turnover pathways, Parkin may mitigate lipid peroxidation and preserve mitochondrial integrity. However, aging and pathological stressors are associated with diminished Parkin expression, which may exacerbate ferroptotic susceptibility and contribute to the progression of myocardial injury^13^.

Importantly, aerobic exercise emerges as a non-pharmacological intervention with the potential to modulate key determinants of ferroptosis. Future research should aim to delineate the precise molecular mechanisms through which aerobic exercise regulates ferroptosis in vivo, with particular focus on the Parkin–ACSL4 axis, p53 signaling, and mitochondrial lipid homeostasis. Targeting these pathways through aerobic exercise or mimetic interventions may pave the way for novel therapeutic approaches in preventing or treating ferroptosis-driven cardiac diseases in the elderly population.

## Limitations and future perspectives

While this review provides new insights into the potential relationship between exercise, ferroptosis, and aging cardiomyocytes, several limitations should be noted. Most current evidence derives from preclinical studies using varied models, exercise protocols, and age ranges, limiting generalizability to human physiology. Standardizing exercise parameters and outcome measures will be essential. Future studies should focus on the in vivo regulation of the Parkin–ACSL4 axis in humans and investigate how exercise can modulate ferroptosis to protect against age-related cardiac dysfunction.

While the proposed Parkin–ACSL4 axis provides a mechanistic framework linking exercise to ferroptosis suppression, current evidence remains largely preclinical. Clinical validation in human populations is still lacking, particularly regarding direct measurements of Parkin activation and ACSL4 ubiquitination in the aging heart. Moreover, the heterogeneity of exercise protocols (type, duration, and intensity) limits the translation of animal findings to clinical guidelines. Future work should employ longitudinal human studies integrating molecular imaging, genetic screening, and metabolomic profiling to establish the therapeutic feasibility of targeting this pathway. If validated, exercise-based interventions could serve as a non-pharmacological strategy to mitigate age-related cardiomyopathy through ferroptosis regulation.

## Data Availability

Data sharing is not applicable to this article as no datasets were generated or analysed during the current study.
